# Prevalence of Constitutional Pathogenic Variant in a Cohort of 348 Patients With Multiple Primary Cancer Addressed in Oncogenetic Consultation

**DOI:** 10.1002/mgg3.70086

**Published:** 2025-03-01

**Authors:** Mathis Lepage, Nancy Uhrhammer, Ioana Molnar, Maud Privat, Flora Ponelle‐Chachuat, Mathilde Gay‐Bellile, Yannick Bidet, Mathias Cavaillé

**Affiliations:** ^1^ Département d'Oncogénétique Centre Jean Perrin Clermont‐Ferrand France; ^2^ INSERM, U1240 Imagerie Moléculaire et Stratégies Théranostiques Université Clermont Auvergne Clermont‐Ferrand France; ^3^ Division de Recherche Clinique Délégation Recherche Clinique & Innovation, Centre Jean PERRIN Clermont‐Ferrand France; ^4^ Département d'Oncogénétique CHU de Québec–Université Laval Quebec Canada

**Keywords:** genetic testing, germline, multiple primary cancer

## Abstract

**Introduction:**

Multiple primary malignancies (MPMs) refer to two or more primary malignant tumors in the same patient. MPMs are frequent: 18.4% of incident cancers represent a second or a higher primary cancer. In order to assess the value of genetic testing for patients with multiple cancers, studies are needed to accurately determine the prevalence of pathogenic variants for these patients.

**Methods:**

All families were seen in our oncogenetics consultation from 2010 to 2022. We compared clinical features and detection rates of pathogenic or likely pathogenic variants in a panel of up to 47 cancer predisposition genes in patients with ≥ 2 primary cancers (*n* = 348) versus a single primary cancer (*n* = 1422).

**Results:**

A pathogenic or likely pathogenic variant was diagnosed in 27.3% of patients with 348 index patients with MPM, concerning 21 genes: *BRCA1* (*n* = 27), *BRCA2* (*n* = 19), *MSH2* (*n* = 9), *ATM* (*n* = 8), *MLH1* (*n* = 5), *MSH6* (*n* = 6), *TP53* (*n* = 4), *CHEK2* (*n* = 4), *PALB2* (*n* = 3), *APC* (*n* = 2), *MEN1* (*n* = 1), *RAD51C* (*n* = 1), *NBN* (*n* = 1), *EPCAM* (*n* = 1), *PMS2* (*n* = 1), *RB1* (*n* = 1), *PTEN* (*n* = 1), *CYLD1* (*n* = 1), *NF1* (*n* = 1), *RAD51D* (*n* = 1), and *CDKN2A* (*n* = 1). MPM index cases were more likely to carry a deleterious mutation than cases with a single cancer (27.3% vs. 11.39%, *p* < 0.001). Pathogenic variants were found more frequently in patients with a suggestive family history (34.2% vs. 20.1%, *p* < 0.05), with a younger age of cancer diagnosis related to the suspected syndrome (32.7% vs. 22%, *p* = 0.049). For the 208 index patients with ≥ 2 cancers pertaining to the same predisposition syndrome (HBOC, HNPCC…), the detection rate increased significantly to 36% (vs. 14.3% for MPM patients with unrelated cancers (*n* = 140), *p* < 0.001). Conversely, the detection rate for patients with unrelated cancers was not statistically different from the single‐cancer population (14.3%–11.39%, *p* = 0.318).

**Conclusion:**

Patients referred for oncogenetic testing with MPM are more likely to carry pathogenic variants in cancer predisposition genes than patients with a single primary cancer (*p* < 0.05), especially if the cancers are related to the same predisposition syndrome. If the cancers are unrelated, no statistical difference in comparison to the single‐cancer population was observed. For these latter patients, we recommend using the specific criteria of each tumor to propose appropriate genetic testing.

## Graphical abstract

Patients recruited from the oncogenetic department with multiple cancers (348) were more likely to carry pathogenic mutations than those with a single cancer (1422) (27.3% vs. 13.39% *p* < 0.001). However, if the multiple primary cancers cannot be linked to the same hereditary predisposition syndrome, the prevalence of pathogenic variants is not significantly different from patients with a single tumor (14.39%–11.39%, *p* = 0.318). In our study, the number of cancers is therefore not a sufficient argument for genetic testing.

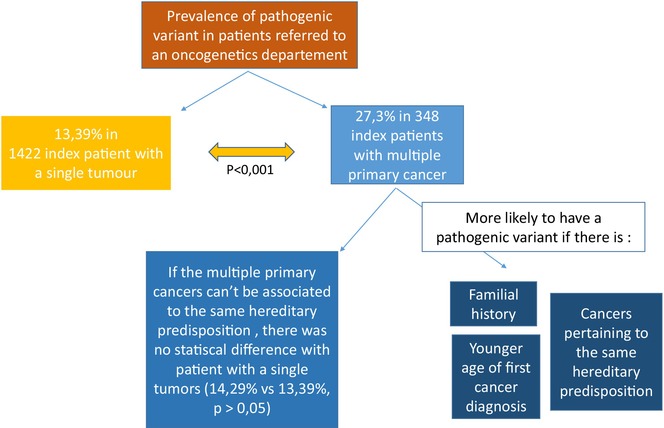

## 1 Introduction

Initially reported by Billroth in 1889 (Billroth 1889), multiple primary cancers are currently defined by the Surveillance Epidemiology and End Results (SEER) and the International Association of cancer Registries (IACR) as more than one malignancy if they arise in different sites and/or are of a different histology (Adamo et al. 2018; Working Group Report&NA 2005). This avoids misclassification of multifocal/multicentric tumors or metastases as multiple primaries. The incidence of MPM is growing as the number of people surviving after cancer diagnosis and the length of their survival period are both increasing year by year, thanks to improvements in early detection, supportive care, and treatment efficiency (America cancer Society 2019). Approximately one‐fourth of older (≥ 65 years) and 11% of younger adults newly diagnosed with cancer had a history of prior cancer in overall cases of incident cancers diagnosed between 2009 and 2013 in the USA (Murphy et al. 2018).

The risk of a subsequent cancer in previously diagnosed individuals is higher than the expected population risk of cancer (Supramaniam 2008; Jégu et al. 2014). This increased risk may be due to a variety of factors, including cancer predisposition syndromes, environmental exposures, and late effects of therapies (Vogt et al. 2017). Since patients with MPM cancers have worsened overall survival (Keegan et al. 2017), deeper insight into the risk factors is needed to improve their prognosis.

It's estimated that 5%–10% of cancers arise due to hereditary predisposition. This proportion is higher for multiple primary cancers (Garber and Offit 2005). Indeed, occurrence of some identified multiple primary tumors was reported as marker of genetic susceptibility. For instance, occurrence of breast and ovarian cancers suggest a pathogenic variant in *BRCA1* (OMIM: 604370) and *BRCA2* (OMIM: 612555): it's an indication to perform a genetic analysis. However, little is known about multiple primary cancers and genetic susceptibility, regardless of cancer type and location. Few studies estimated the prevalence of pathogenic variants for these patients when addressed in the oncogenetic department. A pathogenic or likely pathogenic variant was detected between 13.6% and 35.5% (Bychkovsky et al. 2022; Whitworth et al. 2018; Chan et al. 2018).

Major indications to perform genetic tests are tumor phenotype, family medical history, and patient age at cancer diagnosis. There is no indication based on the number of cancers regardless of cancer type or location. As Whirtworth et al. suggested, if an MPM patient addressed in the oncogenetic department has a pathogenic variant in more than 30%, multiple primary cancers should be an indication to perform genetic tests.

But despite also a high positive detection rate reported by Chan et al., no statistical difference was observed between patients with multiple primary cancers addressed in oncogenetic consultation and those with a single tumor. Such observation strengthened the relevance of carrying out investigations to confirm the detection rate and clarify the indications for genetic testing.

In this study, we investigated whether positive detection of pathogenic variants in cancer susceptibility genes was enriched for patients with multiple primary cancers compared to those with a single cancer.

## 2 Materials and Methods

### 2.1 Ethical Statement

The study has been approved by the local ethics committee (2020/CE 55).

### 2.2 Patients Cohort

#### 2.2.1 Inclusion Criteria

Patient were recruited from the oncogenetic department of the Center Jean Perrin in France from 2010 to 2022. Patients were included if they had multiple primary cancers (excluding nonmelanoma skin cancer), synchronous or metachronous. As recommended by the IACR, the length of time between two cancers was not a criterion for multiple primary cancer definition (homolateral recurrent breast cancer even after 5 years was counted as only one primary cancer). Multiple cancers at one site were counted once except for bilateral breast cancer and cancers arising in different parts of the colon (ascending, transverse, descending and sigmoid colon). Patients with benign histology, metastases, recurrence of the primary tumor of the same site, and/or histological type were excluded.

#### 2.2.2 Assessment of the Prevalence of Pathogenic Variant

The prevalence of pathogenic or likely pathogenic (P/LP) variants was estimated for all patients, then stratified by familial history, age of the first cancer, and multiple primary cancers pertaining to the same hereditary predisposition.

The family history was reported up to the second degree regardless of the cancers and extended to the third degree if cancer in the third degree was associated with the suspicion of hereditary cancer syndrome. A suggestive family history was defined by the presence of at least two cancers in the same family branch or cancer at an abnormally young age (< 50) relevant to the spectrum of the suspected hereditary predisposition. The only exceptions were ovarian cancer and sarcomas, for which a single cancer was sufficient for a suggestive history of a suspected breast/ovarian syndrome and Li‐Fraumeni syndrome, respectively. As only a few cases of familial sarcoma were present, there was no age limit.

Primary multiple cancers were considered pertaining to the same predisposition syndrome if at least two cancers could be associated with the same hereditary syndrome, and this reinforced the indication for a genetic analysis (ex: breast and ovarian cancers). They were considered as unrelated if none of the cancers could be related to the same hereditary predisposition or the presence of these different cancers did not influence the indication for genetic analysis (ORL cancer and breast cancer) (Table 1) For breast and thyroid cancers involved in *PTEN*‐related syndrome, additional clinical features were required, such as macrocephaly and skin signs, to estimate the clinical score (Pilarski et al. 2013). If there were no indications of a genetic test using the clinical score, the two cancers were not considered to be associated.

**TABLE 1:** Most frequent related/unrelated cancers by indication.

Indication	Related cancer	Unrelated cancer
HBOC	Breast and ovarian cancer Bilateral breast cancer Breast and prostatic cancer Breast and pancreatic cancer Lobular breast cancer and diffuse gastric cancer	Breast cancer and endometrial cancer
TP53 related syndrome	Sarcoma and other cancers Child medulloblastoma and glioblastoma Breast cancer < 31 and hematopoietic cancer	Breast cancer > 31 and hematopoietic cancer
HNPCC	Two tumors in HNPCC spectrum: colorectal, endometrial, stomach, ovarian, pancreas, ureter and renal pelvis, biliary tract, and brain tumors, sebaceous gland adenomas and carcinoma of the small bowel	
Multiple endocrine neoplasia	Multiple neoplasia endocrine tumors (thymic carcinoma and malignant pancreatic endocrine tumor)	
*MITF*	Melanoma and renal tumors	
*PTEN* related syndrome	Two tumors of the *PTEN* syndrome (Breast, thyroid, endometrial colon and renal cancer) and indication of genetical analysis using *PTEN* clinical score	Two cancers of *PTEN*‐syndrome and no indication of analysis using *PTEN* clinical score

Patients were then divided into five indications:

– Breast and ovarian cancer
– Digestive cancer (including colorectal cancer, endometrial cancer, stomach cancer and at least two cancers of the hereditary nonpolyposis colorectal cancer (HNPCC)‐related tumors)
– Renal cancer
– Sarcoma
– Other

Patient included in an indication had developed at least one cancer of the indication and had undergone relevant analysis for pathogenic variant detection. A patient can be counted in more than one indication (patient with a breast and a colorectal cancer were included in HBOC and digestive cancer if breast cancer and colorectal predisposing gene were analyzed).

#### 2.2.3 Control Population

**FIGURE 1:**
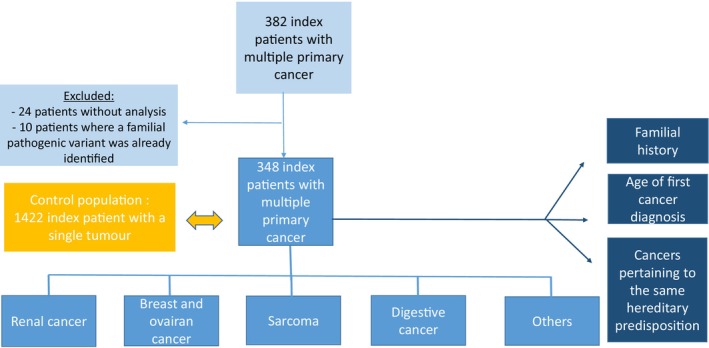
Flow chart of the study. Once the prevalence of pathogenic variants was known for all patients, the prevalence was estimated based on family history, age of the first cancer diagnosis, and multiple primary cancers pertaining to the same hereditary predisposition and then by indication. The detection rate was compared to 1422 patients with a single tumor from our oncogenetic consultation.

The detection rate of pathogenic and probably pathogenic (P/PP) variants in 1530 patients referred to our oncogenetics department between 2016 and 2018 was published in 2021 (Cavaillé et al. 2021). Of these 1530 patients, we excluded all patients with multiple primary cancers. The prevalence of P/PP variants was calculated for the 1422 index patients with a single tumour and used as a reference value (Figure 1).

### 2.3 Sequencing

DNA was extracted from peripheral blood using QIAamp DNA Blood maxikit (Qiagen).

Before 2014, genetic testing comprised oriented gene testing with Sanger sequencing and deletion/duplication analysis of suspected syndromes. Sanger sequencing was performed using a 3500×L instrument and BigDye terminator kit 3.1 (Applied Biosystems). Interpretation was performed using Seqman software (DNASTAR). Copy number variation (CNV) was confirmed using QMPSF (quantitative multiplex PCR of short fragments) or MLPA (Multiplex Ligation‐dependent Probe Amplification).

After 2014, next‐generation sequencing (NGS)‐based multi‐gene panel testing, including up to 47 genes, was performed (Table 2). Sonic fragmentation of DNA from peripheral blood was performed on a Bioruptor instrument (Diagenode). Kapa HTP library preparation and SeqCap EZ Choice probes and reagents (Roche) were used for library preparation and capture. The quality of fragmentation, library, and capture was controlled using a Bioanalyzer 2100 instrument (Agilent). Sequencing was performed using the Miseq v2 kit (300 cycles) on the Miseq Instrument (Illumina). All steps were performed following providers' guidelines. Analysis of exons 11 to 15 of PMS2 and exons 1, 13, and 14 of SDHA was not performed due to high identity with paralog genes. Any exons with insufficient coverage depth (<  50×) in genes pertinent to the clinical presentation were analyzed for point mutations by Sanger sequencing and for CNV by QMPSF as described above.

**TABLE 2:** Diagnostic genes.

Gene	OMIM number	Reference	Gene	Reference	Gene	OMIM number	Reference
AIP	605555	LRG_460	FLCN	NM_144997.5	RAD51C	602774	NM_058216.2
APC	611731	NM_000038.5	MAX	NM_002382.4	RAD51D	602954	NM_002878.3
ATM	114480	NM_000051.3	MEN1	NM_130799.2	RET	164761	NM_020975.4
BAP1	603089	NM_004656.3	MET	LRG_662	SDHA	600857	NM_004168.2
BMPR1A	601299	NM_004329.2	MITF	LRG_776	SDHAF2	613019	NM_017841.2
BRCA1	113705	NM_007294.3	MLH1	NM_000249.3	SDHB	185470	NM_003000.2
BRCA2	600185	NM_000059.3	MSH2	NM_000251.2	SDHC	602413	NM_003001.3
BRIP1	605882	NM_032043.2	MSH6	NM_000179.2	SDHD	602690	NM_003002.3
CASR	601199	NM_000388.3	MUTYH	NM_001048171.1	SMAD4	600993	NM_005359.5
CDC73	607393	LRG_507	NBN	NM_002485.4	STK11	602216	NM_000455.4
CDH1	192090	NM_004360.3	NF1	NM_000267.3	TMEM127	613403	NM_017849.3
CDK4	123829	LRG_490	NF2	NM_000268.3	TP53	191170	NM_000546.5
CDKN2A	600160	NM_000077.4 et NM_058195.3	PALB2	NM_024675.3	VHL	608537	NM_000551.3
CHEK2	604373	NM_007194.3	POLD1	NM_001256849.1	WRN	277700	NM_000553.4
EPCAM	185535	NM_002354.2	POLE	NM_006231.2			
FH	136850	LRG_504	*PTEN*	NM_000314.4			

If there was a suspicion of a hereditary cancer syndrome not explored in our center, the analysis was outsourced to a reference laboratory.

### 2.4 Bio‐Informatic Analysis

De‐multiplexing was performed using bcl2fastq2 Conversion Software (Illumina). Alignment was performed on University of California Santa Cruz human genome reference build 19 using the Burrows‐Wheeler Aligner. Genome Analysis Toolkit (GATK) and PICARD tools were used for base quality score recalibration (BaseRecalibrator) and realignment (RealignerTargetCreator, IndelRealigner), as recommended by Eurogentest guidelines (Matthijs et al. 2016). Variant calling was performed using GATK HaplotypeCaller and annotated using Ensembl Variant Effect Predictor. CNV analysis was performed using ExomeDef. Variants were filtered for quality score ≥ 30, depth ≥ 50×, and present in ≥ 20% of reads.

### 2.5 Variant Interpretation

Variant interpretation was performed using ALAMUT (Interactive BioSoftware), which includes splice site analysis tools (SpliceSiteFinder, MaxEntScan), protein‐function prediction tools (SIFT, Polyphen 2.0), and links to relevant databases (ClinVar, Leiden Open Variation Database [LOVD], other syndrome‐specific databases). Variants were classified according to the American College of Medical Genetics (ACMG) recommendations (Richards et al. 2015), aided by the French National Database of variants (Groupe Génétique et Cancer). Only patients with class 4 or 5 variants that lead to recommendations for health management were considered as positive (Biallelic MUTYH were considered a PV, and monoallelic MUTYH was not).

### 2.6 Statistical Analyses

The difference between the control population and patients with multiple primary cancers was tested using the exact binomial test, with comparisons between categories using Fisher's exact test. The *p*‐value was adjusted for multiple tests (Benjamini‐Hochberg type correction). All statistical tests were two‐sided and used a 5% significance level.

## 3 Results

### 3.1 Epidemiology

Three hundred forty‐eight patients (45 [13%] males and 303 [87%] females) had been diagnosed with multiple primary cancers (815 primary cancer) (Table 3). The prevalence of multiple primary cancers was 8.2% in our oncogenetic department. The main indications for multiple primary cancer were similar to oncogenetic consultations (Cavaillé et al. 2021):

– Major indication breast/ovary (HBOC): 84% (population control 76%, 71% of consultations across France (Institut National Du Cancer n.d.))
– Digestive pathologies (Lynch syndrome, polyposis): 23% (population control 15%, 17% in France)
– Endocrine pathologies: 1.4% (population control 5%, 1.9% in France)

**TABLE 3:** Patient demographics.

	Global analysis
Index patient	348
Women	303 (87%)
Men	45 (13%)
Age of first cancer	50
Age of second cancer	60
Pathology report available	83%
Patients with more than 10 genes analysed	61%

**FIGURE 2:**
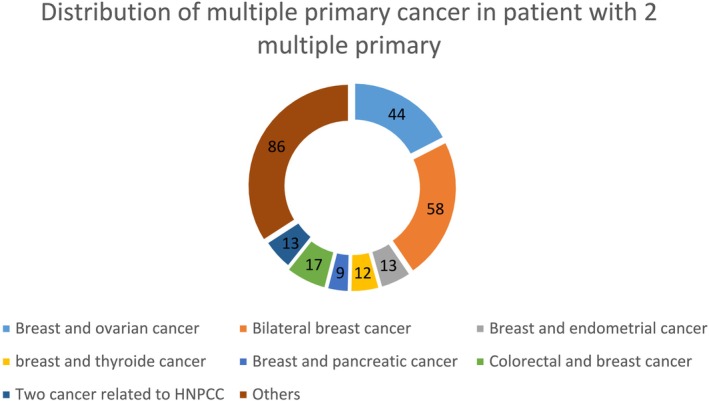
Only multiple primary cancers reported more than eight times were individualized. A third of the multiple primary cancers are associations that occurred less than nine times.

For the patients with two primary cancers (*n* = 252), the most common cancer pairs were bilateral breast (*n* = 58), breast–ovary (*n* = 44), breast–colorectal (*n* = 17) and breast–endometrium (*n* = 13) (Figure 2).

### 3.2 Prevalence and Distribution of Pathogenic or Likely Pathogenic Variant

Overall, 27.3% (*n* = 105) of tested patients with multiple primary cancers were found to carry a pathogenic or likely pathogenic variant. The difference was statistically significant compared to the single‐cancer population (27.3 vs. 11.39%, *p* < 0.001). The P/PP variants were identified in 21 genes: *BRCA1* (*n* = 27), *BRCA2* (*n* = 19), *MSH2* (*n* = 9), *ATM* (*n* = 8), *MLH1* (*n* = 5), *MSH6* (*n* = 6), *TP53* (*n* = 4), *CHEK2* (*n* = 4), *PALB2* (*n* = 3), *APC* (*n* = 2), *MEN1* (*n* = 1), *RAD51C* (*n* = 1), *NBN* (*n* = 1), *EPCAM* (*n* = 1), *PMS2* (*n* = 1), *RB1* (*n* = 1), *PTEN* (*n* = 1), *CYLD1* (*n* = 1), *NF1* (*n* = 1), *RAD51D* (*n* = 1), and *CDKN2A* (*n* = 1). Three patients had two pathogenic variants (*APC* and *ATM*, *BRCA1* and *RAD51C*, *BRCA1* and *NBN*). P/LP variants could explain all the cancers in 60% of patients, while in 40% at least one cancer could not be attributed to hereditary predisposition.

**FIGURE 3:**
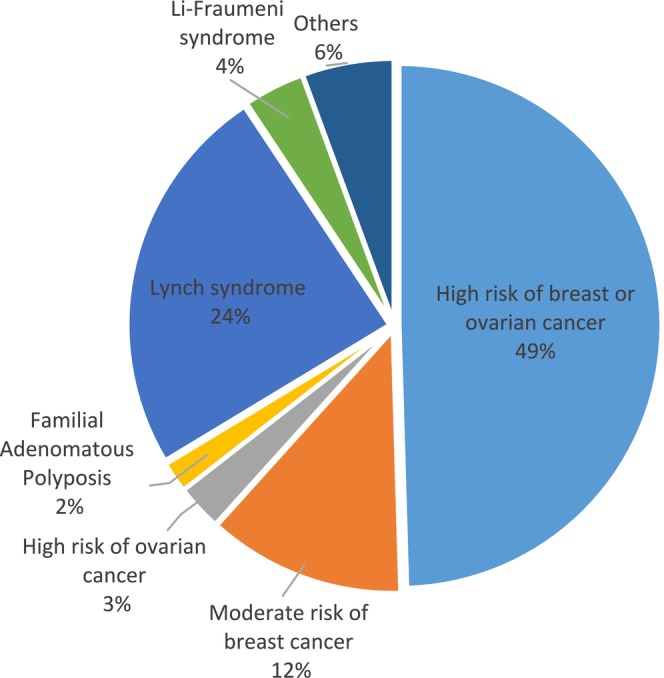
Hereditary cancer syndrome identified. High risk of breast or ovarian cancer (*BRCA1, BRCA2, PALB2*) represents 49% of the hereditary cancer syndromes identified, moderate risk of breast cancer (*ATM, CHEK2, NBN*) 12%, and high risk of ovarian cancer (*RAD51C, RAD51D*) *3*%.

Hereditary predisposition to breast and/or ovarian (HBOC) cancer indication accounts for 65% of the identified pathogenic variants (*BRCA1*, *BRCA2, PALB2*, *ATM*, *CHEK2*, *NBN*, *RAD51C*, and *RAD51D*); Lynch syndrome (*MLH1*, *MSH2*, *EPCAM*, *PMS2* and *MSH6*) represented 24.3%, and familial adenomatous polyposis (*APC*) 1.9% of the variants (Figure 3).

We found in approximately 1% an incident pathogenic variant (three pathogenic variants among MSH6, ATM, and CHEK2) which is comparable to the prevalence of incident mutations (from 1% to 2%) in the literature or among the control population (Amendola et al. 2015).

#### 3.2.1 Prevalence of Pathogenic Variant by Familial History, Age, Number of Cancers, and Cancers Pertaining to Same Hereditary Predisposition

Patients with a suggestive family history of cancer included in the primary suspected cancer syndrome were more likely to carry a deleterious variant (34.44% vs. 20.12%, *p* = 0.02). Even when there was no suggestive familial history, patients with multiple primary cancers were more likely diagnosed with deleterious mutations than those with single primary cancers (20.12% vs. 11.39%, *p* = 0.002).

Positive detection rate was not correlated to the age of first cancer (cancer before ≤ 50:29.19% (*n* = 185), > 50:24.54%, *p* > 0.354). But if the threshold was applied to the cancer related to the suspicion of hereditary cancer predisposition, a statistical difference was then observed (≤ 50:32.72% (*n* = 162), > 50:22.04% (*n* = 186), *p* = 0.049).

Patients who reported two cancers (252 patients) were diagnosed with a pathogenic variant in 24.2% of cases, patients with three cancers (78 patients) in 33.3% of cases, and patients with more than three cancers (18 patients) in 44.4% of cases.

A pathogenic variant was identified in 36.06% of cases among patients with multiple cancers pertaining to the same hereditary predisposition (HBOC, HNPCC…). Otherwise, the prevalence was only 14.29%. The diagnostic rate of patients with unrelated cancers was not statistically different from the control population (14.29%–11.39%, *p* > 0.05).

#### 3.2.2 Prevalence of Pathogenic Variant by Indication and Cancer Localization

The prevalence of P/LP variant was:

−23.5% in the breast/ovary cancer indication among 298 patients. The difference is significant (*p* < 0.001) compared to the control population analyzed for this indication (10.96%). Among this indication, only patients with breast and ovarian cancer and patients with bilateral breast cancer had a significant difference with the single‐cancer population (P/LP variant in 24.71% vs. 10.96%, *p* = 0.001 and 39.66% vs. 10.96%, *p* < 0.001).

– 35.8% in digestive cancer indication among 80 patients (*p* < 0.001 compared to the control population which presented the same indication (14.95%)). Among this indication, only patients with at least two cancers pertaining to Lynch syndrome had a significant difference with the single‐cancer population (64.0% vs. 14.95%, *p* < 0.001).
– 50% in the case of sarcoma (14 patients), 9% in kidney cancer (22 patients) and 36.7% in other indications (including multiple neoplasia syndrome, retinoblastoma …) (19 patients).

## 4 Discussion

Multiple primary cancer is currently a problem of public health: the incidence of multiple primary cancer is rising, and their prognosis is worse than that of a single tumor of the same histology. In order to offer a personalized follow‐up to patients, it is necessary to deepen our knowledge of the risk factors associated with multiple primary cancer. Although suggested by a few studies, there is currently no recommendation for genetic testing based on the number of tumors regardless of cancer type.

We described in this study a cohort of 348 index patients with MPM who underwent genetic testing. Pathogenic or likely pathogenic variants were identified in 27.3% of index patients, in 21 genes. Patients with multiple primary cancers were more likely to carry pathogenic variants than those with single primary cancers (27.3% vs. 11.39%, *p* < 0.01).

In previous reports, the prevalence of P/LP variants in index patients with MPM, regardless of cancer type, ranges from 13.6% to 35.5% (Bychkovsky et al. 2022; Whitworth et al. 2018; Chan et al. 2018).

– Chan et al. identified a pathogenic variant in 35.5% of patients with multiple primary cancers (*n* = 110) who underwent genetic analysis using a multi‐gene panel comprising up to 49 genes. There was no statistical difference with single cancer patients (*p* = 0.09). The prevalence of pathogenic variants was very high in the group of single cancer patients (25.6%) in comparison to reported studies (8%–15%) and to our control population. It may explain that the difference did not reach the statistical threshold (LaDuca et al. 2020; Susswein et al. 2016; Neben et al. 2019).
– Whirtworth et al. estimated that comprehensive genetic analysis of individuals (83 genes) with MPTs (and no prior genetic testing) addressed in genetic centers would detect a pathogenic or likely pathogenic variant in around a third of individuals (32.8%) from two studies of 212 patients and 460 patients (Whitworth et al. 2015, 2018). Multiple primary tumors were defined as at least two primaries by age 60 years or at least three by 70 years. There was no control population.
– In a cohort of more than 9000 patients with primary multiple cancers, a pathogenic variant was identified in 13.6% of cases among 21 genes (ATM, BARD1, BRIP1, CHEK2, MSH2, MSH6, NBN, NF1, PALB2, PMS2, PTEN, RAD51C, RAD51D, and TP53). There was no control population, so it was not possible to compare the prevalence of pathogenic variants in patients with multiple primary cancers or with a single cancer.

Patients with P/LP variants in cancer predisposition genes were more likely to have a suggestive family history (34.44% vs. 20.12%, *p* = 0.02) and were younger at cancer diagnosis related to the suspicion of hereditary cancer predisposition (≤ 50:32.72%, > 50:22.04%, *p* = 0.049). Suggestive family history and younger age are two well‐known risk factors of hereditary cancer predisposition (Momozawa et al. 2018). The prevalence of P/LP variants was not correlated with the age of cancer diagnosis if all first cancers were considered (cancer related and unrelated to the suspicion of hereditary cancer predisposition). Maxwell et al. observed the same result in a cohort of breast cancers and a second primary cancer, where the age of the other cancer was not correlated with the presence of P/LP variants in a breast cancer predisposing gene (Maxwell et al. 2020). It could suggest that cancers unrelated to hereditary predisposition were not influenced by the presence of P/LP variants.

Patients with multiple primary cancers without a family history present in 20% a P/LP variant. Whirtworth et al. also noted a large proportion of patients with multiple primaries who had a P/LP variant without a family history of cancer in a first‐degree relative (Whitworth et al. 2018). Absent of family history should therefore not be a sufficient criterion to exclude a genetic analysis in these patients.

The prevalence of an inherited cancer predisposition syndrome was 36% when at least two cancers belonged to the same hereditary predisposition and only 14.3% when there was no evident association. The prevalence of pathogenic variants was not significantly different in patients with unrelated multiple cancers and the control population (14.3%–11.39%, *p* = 0.318). Despite the significant increase in the prevalence of pathogenic variants identified in multiple cancers, the number of cancers is not sufficient to justify a genetic analysis.

Primary multiple cancers could also arise from patients with multilocus inherited neoplasia allele (MINA) syndrome. In our cohort, three patients were diagnosed with two pathogenic variants (*BRCA1* and *NBN*, *ATM* and *APC*, *BRCA1* and *RAD51C*). The first patient had developed two breast cancers at 27 and 48 years old, the second patient a breast cancer and a polyposis. The last patient presented with a breast cancer at 50 and a pancreatic cancer at 64 but did not have any ovarian cancer until 65 despite two pathogenic variants predisposing her to ovarian cancer. As described in a review of patients with MINA, the interaction between two pathogenic variants in different loci is not readily predictable but is indeed a rare cause of multiple primary cancers (Whitworth et al. 2016).

At least one cancer could not be attributed directly to a hereditary predisposition in 40% of patients with P/LP variants in our study. The association between pathogenic variants and the cancer was established on the known spectre of the pathogenic variant (e.g., *BRAC2*: breast, ovarian, prostate and pancreatic cancer). We did not analyze tumor tissues to search for a double hit in the non‐associated cancer. It would be interesting, but it was not possible as few patients were treated for their multiple primary cancers in our hospital, and some tumors were no longer available.

Sporadic cancers also occur in patients with hereditary predispositions; common risk factors such as tobacco must also be evaluated for these patients. Other factors of multiple primary cancer could be observed in this cohort, such as the adverse effect of the treatment of the first cancer and lifestyle risk (Travis et al. 2006). Two patients treated by radiotherapy after a Hodgkin lymphoma developed a breast cancer. About 8% of second solid cancers may be related to the radiotherapy treatment for the first cancer (de Gonzalez et al. 2011). Few patients had multiple primary cancers related to tobacco/alcohol risk (ORL and lung cancer) as they were selected in an oncogenetic department. In the SEER cancer registries 1973–2000, tobacco/alcohol‐related cancer sites accounted for more than 35% of the total excess subsequent cancers (Supramaniam 2008).

One of the limitations of this study is the inclusion from an oncogenic department. As patients referred to the oncogenetic department represent a high‐risk population (type of tumours, family history …), they may not reflect mutation rates in the general population. Gynaecological cancers were over‐represented as they are the major indication of oncogenetic tests, whereas in the general population, the most frequent multiple primary tumours occur in different site (Supramaniam 2008).

Finally, we confirm that patients referred to the oncogenetic department with multiple primary cancers were more likely to carry pathogenic mutations in cancer predisposition genes than those with a single primary cancer (*p* < 0.05). Only patients with multiple primary cancers related to the same hereditary condition had a higher detection rate and must have a genetic test. If multiple primary cancers were unrelated, no difference was observed with the control population. For these patients, we recommend using the specific criteria of each tumor to propose a genetic test.

## Author Contributions

Conceptualization, M.L. and M.C.; software, F.P.‐C.; statistical analysis: I.M.; formal analysis; investigation, M.L., M.C., M.G.‐B.; writing – original draft preparation, M.L.; writing – review and editing, M.L., N.U., M.P., Y.B., M.G.‐B., and M.C.; supervision, M.C.; all authors have read and agreed to the published version of the manuscript.

## Conflicts of Interest

The authors declare no conflicts of interest.

## Data Availability

Data are available from the corresponding author on reasonable request.
